# Homoarginine and creatine deficiency do not exacerbate murine ischaemic heart failure

**DOI:** 10.1002/ehf2.14183

**Published:** 2022-09-30

**Authors:** Debra J. McAndrew, Hannah A. Lake, Sevasti Zervou, Edzard Schwedhelm, Jurgen E. Schneider, Stefan Neubauer, Craig A. Lygate

**Affiliations:** ^1^ Division of Cardiovascular Medicine, Radcliffe Department of Medicine University of Oxford Oxford UK; ^2^ British Heart Foundation Centre for Research Excellence University of Oxford Oxford UK; ^3^ Wellcome Centre for Human Genetics Roosevelt Drive Oxford OX3 7BN UK; ^4^ Institute of Clinical Pharmacology and Toxicology University Medical Center Hamburg‐Eppendorf Hamburg Germany; ^5^ Experimental and Preclinical Imaging Centre (ePIC), Leeds Institute of Cardiovascular and Metabolic Medicine University of Leeds Leeds UK

**Keywords:** Homoarginine, Creatine, Ventricular function, Heart failure, Myocardial infarction, Animal models

## Abstract

**Aims:**

Low levels of homoarginine and creatine are associated with heart failure severity in humans, but it is unclear to what extent they contribute to pathophysiology. Both are synthesized via L‐arginine:glycine amidinotransferase (AGAT), such that AGAT^−/−^ mice have a combined creatine and homoarginine deficiency. We hypothesized that this would be detrimental in the setting of chronic heart failure.

**Methods and results:**

Study 1: homoarginine deficiency—female AGAT^−/−^ and wild‐type mice were given creatine‐supplemented diet so that both had normal myocardial creatine levels, but only AGAT^−/−^ had low plasma homoarginine. Myocardial infarction (MI) was surgically induced and left ventricular (LV) structure and function assessed at 6–7 weeks by *in vivo* imaging and haemodynamics. Study 2: homoarginine and creatine‐deficiency—as before, but AGAT^−/−^ mice were given creatine‐supplemented diet until 1 week post‐MI, when 50% were changed to a creatine‐free diet. Both groups therefore had low homoarginine levels, but one group also developed lower myocardial creatine levels. In both studies, all groups had LV remodelling and dysfunction commensurate with the development of chronic heart failure, for example, LV dilatation and mean ejection fraction <20%. However, neither homoarginine deficiency alone or in combination with creatine deficiency had a significant effect on mortality, LV remodelling, or on any indices of contractile and lusitropic function.

**Conclusions:**

Low levels of homoarginine and creatine do not worsen chronic heart failure arguing against a major causative role in disease progression. This suggests that it is unnecessary to correct hArg deficiency in patients with heart failure, although supra‐physiological levels may still be beneficial.

## Introduction

**Figure 1:**
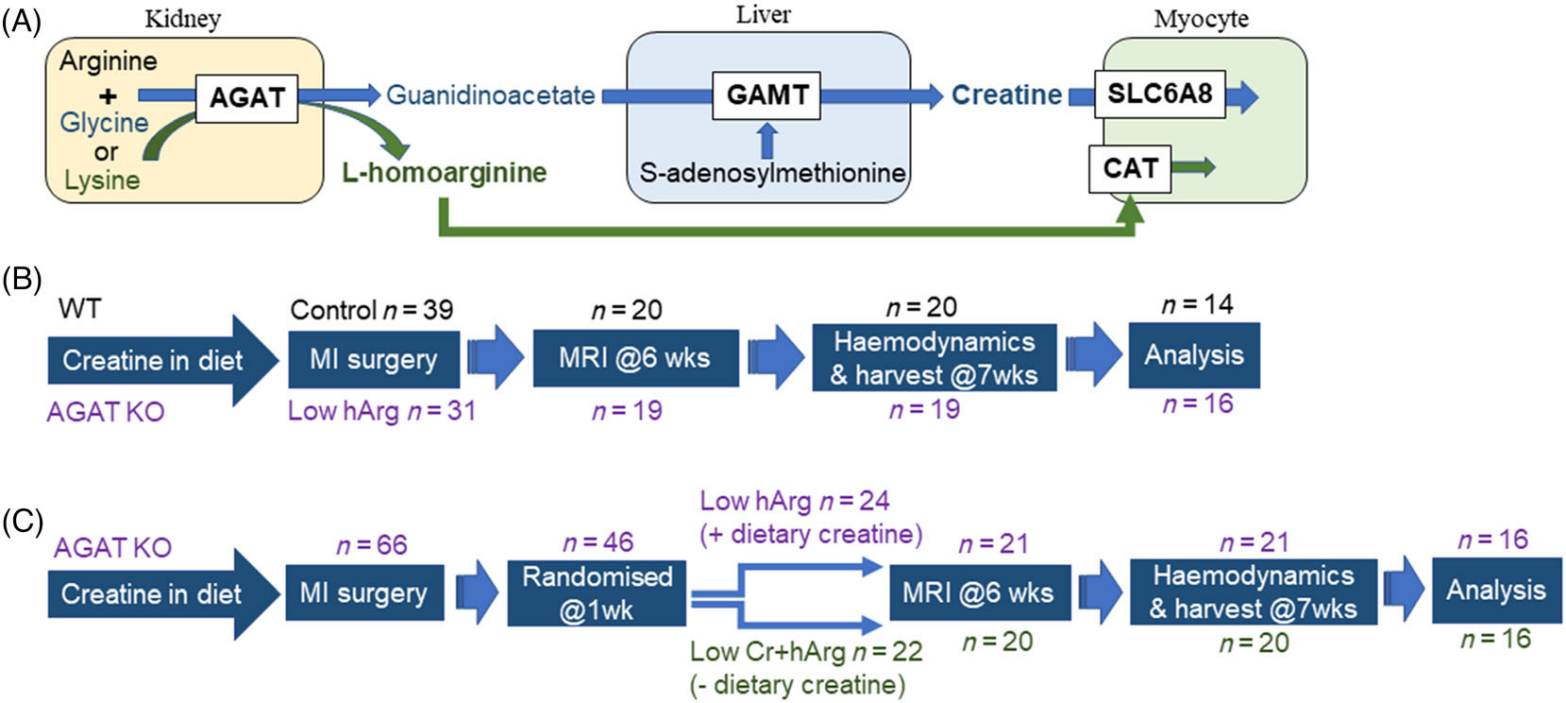
Biosynthetic pathway and study protocol schematics. (A) Biosynthesis of creatine from arginine and glycine by L‐arginine:glycine amidinotransferase (AGAT) and guanidinoacetate methyltransferase (GAMT) is compartmentalized in kidney and liver with cellular uptake via the creatine transporter (SLC6A8). L‐homoarginine (hArg) is also synthesized by AGAT from arginine and lysine and is taken up via the cationic amino acid transporter (CAT). (B) Study 1 protocol: homoarginine deficiency in heart failure. All mice are fed creatine in the diet throughout. (C) Study 2 protocol: homoarginine and creatine‐deficiency in heart failure. AGAT KO mice are fed dietary creatine until 1 week post‐surgery to induce myocardial infarction. They are then randomized to continue on creatine‐diet or are changed to a creatine‐free diet. Exclusions at the analysis stage are due to technical failure (e.g., death during haemodynamics) and exclusions to match groups for infarct size.

Creatine and L‐homoarginine are amino acid derivatives that share a biosynthetic enzyme, arginine:glycine amidinotransferase (AGAT; *Figure* *1* *A*). Low levels of either metabolite are considered biomarkers for heart failure severity, but the extent to which this may contribute to disease pathophysiology remains to be established.

Multiple population‐based studies have identified low circulating homoarginine (hArg) levels as an independent risk factor for adverse outcomes and mortality in cardiovascular disease, including in MI and heart failure.^1^, ^2^ The reason for this association is not obvious because homoarginine (hArg) is a non‐proteinergic amino acid with no essential biochemical function. More pragmatically, for individuals with naturally low levels of circulating hArg, the fundamental question is whether this predisposes them to more severe heart failure.

Creatine is a component of the creatine kinase (CK) phosphagen system that helps the heart respond to high energy demand; however, both creatine levels and CK activity are reduced in the failing heart.^3^ Evidence from cardiomyopathy patients and multiple animal models indicate that these changes correlate strongly with heart failure severity.^3^, ^4^, ^5^, ^6^ However, data demonstrating causation are less convincing. For example, mice lacking the second enzyme in creatine biosynthesis (guanidinoacetate *N*‐methyltransferase; GAMT) exhibit reduced contractile reserve,^7^ but this did not translate into more severe ischaemic heart failure.^8^ The counterargument is that these mice have multiple potential confounders, for example, low body weight, compensatory adaptations to life‐long creatine deficiency, and the accumulation of a semi‐functional creatine precursor.^8^, ^9^ Hence, the question of whether low creatine *per se* helps drive heart failure progression has yet to be adequately resolved.

Herein, we use the AGAT knockout (KO) mouse to address these fundamental questions. AGAT is the first enzyme in creatine (and hArg) biosynthesis, so they do not accumulate creatine intermediates and the metabolite levels can be easily manipulated in the diet to prevent chronic adaptation. Furthermore, AGAT is not normally expressed in the heart, so the absence of protein is not a confounder. These mice have been used to untangle the effects of creatine and hArg deficiency. For example, AGAT KO exhibit very low body weight due to minimal fat deposits, skeletal muscle weakness and atrophy, all of which are fully rescued by dietary creatine.^10^, ^11^, ^12^ AGAT KO hearts also have impaired indices of contractile and lusitropic function, although hallmarks of heart failure are absent.^11^ Surprisingly, dietary supplementation with hArg (but not creatine) rescued these haemodynamic parameters.^11^ We therefore hypothesize that low hArg levels in the setting of chronic heart failure would be detrimental, resulting in impaired *in vivo* function and adverse remodelling.

To this end, we have fed AGAT KO and wild‐type (WT) mice creatine in the diet from birth, such that creatine levels were normal and comparable in both groups. The only key difference was therefore low circulating levels of hArg in the KO mice. These were subjected to MI and the development of heart failure assessed 6 weeks later. This mimics individuals in the general population who are in the lower quartile for circulating hArg and therefore considered at higher risk of major adverse cardiovascular events.^13^

In a follow‐up study, we hypothesize that low creatine and low hArg are synergistically detrimental, that is, that there are low levels of both that drive dysfunction. AGAT KO mice were fed creatine in the diet from birth before inducing a myocardial infarction (MI). Seven days later, half the animals were changed to a creatine‐free diet, in order to exaggerate creatine loss from the heart, but without causing an absolute creatine deficiency. These conditions mimic the situation in patients who have naturally lower levels of circulating hArg, who are then exposed to the characteristic decline in myocardial creatine levels that is a hallmark of the failing heart.

## Methods

### Ethics and animal husbandry

Experiments in mice were approved by the Committee for Animal Care and Ethical Review at the University of Oxford with a licence granted by the Home Office (PPL 30‐3314) under the UK Animals (Scientific Procedures) Act 1986, incorporating European Directive 2010/63/EU. L‐arginine:glycine amidinotransferase knockout mice (AGAT^−/−^) have a homozygous deletion of the *Gatm* ^*tm1.1Isb*^ allele created by homologous recombination, resulting in whole‐body deficiency of both creatine and L‐homoarginine^10^ (n.b. cardiac‐specific KO is unavailable because AGAT protein is not normally expressed in the heart). All procedures used adult mice backcrossed for >10 generations with C57BL/6J^OlaHsd^ and breeding was by inter‐crossing heterozygous animals to produce littermate controls, with genotyping by polymerase chain reaction from ear biopsies as previously described.^10^ Female mice were used throughout for ethical reasons because females have a much lower mortality due to a reduced incidence of cardiac rupture following myocardial infarction.

Mice were housed in specific pathogen‐free cages and maintained on a 12 h/12 h light–dark cycle under conditions of controlled temperature (20–22°C) and humidity (55 ± 10%) with water and chow available ad libitum. Mice were given either R/M‐H complete maintenance diet, which is naturally creatine‐free or the same diet supplemented with 5 g/kg creatine monohydrate (Ssniff, Soest, Germany).

### Study 1 protocol: Homoarginine deficiency in heart failure

1. Female AGAT^−/−^ and WT mice were fed creatine‐supplemented diet throughout their lifetime, such that both had normal myocardial creatine levels, but only the KO had low plasma hArg.
2. Surgery to induce permanent MI at a mean age of 22 weeks for both experimental groups (WT *n* = 39, AGAT^−/−^ *n* = 31).
3. Six weeks post‐MI: cine‐magnetic resonance imaging (MRI) to quantify infarct size, LV volumes, and function.
4. Seven weeks post‐MI: LV haemodynamic assessment at baseline and under maximal β‐adrenergic stimulation using IV dobutamine.
5. Blood sample by cardiac puncture and killing by rapid excision of the heart under deep isoflurane anaesthesia (*Figure* *1* *B*).

### Study 2 protocol: Homoarginine and creatine‐deficiency in heart failure

1. Female AGAT^−/−^ mice were fed creatine‐supplemented diet throughout their lifetime.
2. Surgery to induce permanent MI at a mean age of 22 weeks for both experimental groups (*n* = 66).
3. One week post‐MI: 50% randomized to standard (creatine‐free) diet, such that both experimental groups had low hArg levels, but one group developed lower myocardial creatine levels.
4. Six weeks post‐MI: 3‐D echocardiography to quantify infarct size, LV volumes, and function.
5. Seven weeks post‐MI: LV haemodynamic assessment at baseline and under maximal β‐adrenergic stimulation using IV dobutamine.
6. Blood sample by cardiac puncture and killing by rapid excision of the heart under deep isoflurane anaesthesia (*Figure* *1* *C*).

### Myocardial infarction surgery

Detailed surgical protocols have been described previously.^14^ In brief, general anaesthesia was induced with 4% isoflurane in medical oxygen and 0.08 mL subcutaneous buprenorphine hydrochloride (0.3 mg/mL), with anaesthetic depth assessed by absence of pedal reflex. Oropharyngeal intubation and mechanical ventilation was commenced with isoflurane maintained at 2%. A left intercostal thoracotomy was performed, the pericardium removed, and a 6–0 polyethylene suture placed to permanently occlude the left coronary artery. Mice were given subcutaneous saline and a heated home cage during recovery, with additional 0.04 mL subcutaneous buprenorphine in the evening and following morning.

### MRI and 3‐D echocardiography

Study 1 used cine‐MRI under isoflurane anaesthesia for assessment of LV volumes as previously described.^11^, ^15^ Study 2 used 3‐D echocardiography for assessment of LV volumes because our MRI unit was no longer operational. We have previously validated 3‐D echocardiography against cine‐MRI in the myocardial infarction model and published detailed methods.^16^ Here we used a VisualSonics Vevo2100 with 22–55 MHz transducer (MS 550D) with isoflurane anaesthesia maintained at 1–1.5% throughout. For in vivo determination of infarct size, end‐diastolic contours were traced along the midline circumference on the 3D‐echo and MRI short‐axis slices; only akinetic and dyskinetic segments were considered infarcted. Infarct segment length and LV circumference were summated for all slices and infarct size expressed as a percentage of total LV circumference.

### Haemodynamics and tissue harvest

LV and jugular vein cannulation for haemodynamic assessment were as described previously,^7^ using 4% isoflurane for induction and 1.5–2% for maintenance with anaesthetic depth assessed by pedal reflex. Dobutamine hydrochloride was administered at 32 ng/g BWt/min. Blood was collected via cardiac puncture into lithium heparin tubes, the heart rapidly excised, washed in saline and blotted, the infarct scar removed, and the remaining LV snap frozen in liquid nitrogen. Samples were stored at −80°C until used.

### Biochemistry

#### Myocardial creatine quantification

Frozen LV tissue was powdered and total creatine (i.e., the sum of free creatine and phosphocreatine) was quantified by high‐performance liquid chromatography.^8^

#### Plasma hArg quantification

A subset of plasma samples was used for measurement of circulating hArg levels. Samples from Study 1 were quantified using a stable isotope dilution assay for tandem‐mass spectrometry as previously described.^17^ Samples from Study 2 were quantified using a commercially available Homoarginine ELISA kit as per the manufacturer's instructions (Reference code EA205/96, DLD Diagnostika GBMH, Hamburg, Germany). There was good agreement between these methods.

### Data analysis

These studies were designed to determine the progression to heart failure rather than any putative effect on myocardial injury. This approach requires retrospective matching of infarct size between experimental groups because infarct size is the largest single determinant of dysfunction post‐MI. In addition, mice with infarct size <25% were excluded because they do not develop hallmark signs of heart failure.^18^ All measurements were performed blind to the experimental groups. Survival data were plotted as Kaplan–Meier survival curves and compared using the log‐rank (Mantel–Cox) test. An unpaired two‐tailed Student's *t*‐test was used for all comparisons, with the exception of a Welch's correction where variances were found to be unequal, or a Mann–Whitney test if data had a non‐Gaussian distribution (the test used is indicated in the figure legends). *P* < 0.05 was considered significant and all data was analysed and plotted using GraphPad Prism version 9.3, with post‐hoc power calculations performed using GraphPad StatMate 2.00.

## Results

### Study 1: Homoarginine deficiency in heart failure

**Figure 2:**
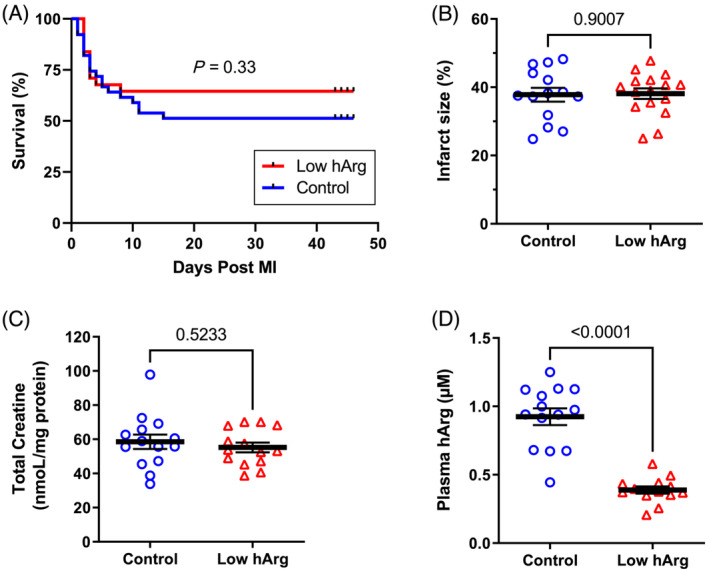
Homoarginine deficiency study: (A) Kaplan–Meier survival curves following myocardial infarction in wild‐type control mice (*n* = 39) and knockout mice with low levels of plasma homoarginine (low hArg; *n* = 31). (B) Surviving mice were matched for infarct size calculated as percentage of LV circumference measured by magnetic resonance imaging (MRI). This resulted in *n* = 14 control and n = 16 low hArg for subsequent analysis with a mean infarct size of 38%. (C) Myocardial levels for total creatine were not significantly different between groups, whereas knockout mice were confirmed to have significantly lower levels of plasma hArg (D). Data are mean ± SEM with log‐rank test to compare survival curves and unpaired Student's *t*‐test for all other comparisons except plasma hArg where a Welch's correction was used to account for unequal variances.

A total of *n* = 39 WT and *n* = 31 KO mice survived surgery and had evidence of a myocardial infarction. Survival curves are shown in *Figure* *2* *A* with mice defined as having ‘died’ if they were found dead or if adverse effects triggered the humane endpoints. These included respiratory distress, body weight loss of 20%, and non‐responsiveness. A log‐rank test indicates that the curves were not significantly different (*P* = 0.33).

To determine the effect on heart failure independent of myocardial injury, data from four WT and three KO mice were excluded to retrospectively match for infarct size, resulting in group sizes of *n* = 14 WT and *n* = 16 KO, both with mean infarct size of 38% and a range from 25–48%. As expected, the addition of dietary creatine to chow meant there was no difference in total creatine levels in the heart. However, plasma hArg levels were significantly lower in knockout animals with a mean value that was 42% of control levels (*Figure* *2*).

**Figure 3:**
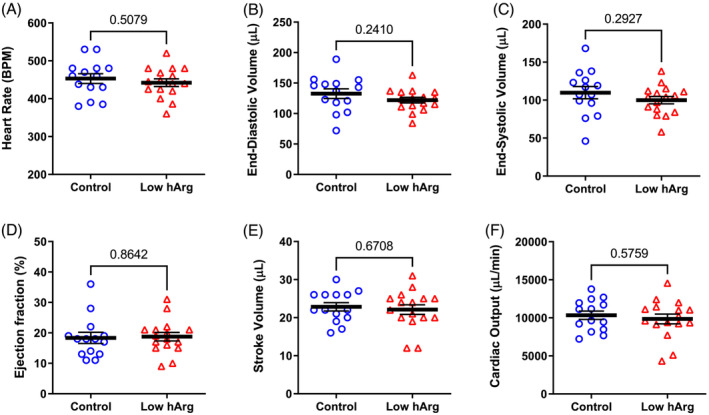
Homoarginine deficiency study: cardiac cine‐magnetic resonance imaging (MRI) at 6 weeks after myocardial infarction. (A) Heart rate; (B) LV end‐diastolic volume; (C) LV end‐systolic volume; (D) ejection fraction; (E) stroke volume; (F) cardiac output. Data are mean ± SEM from *n* = 14 control and *n* = 16 low hArg mice using unpaired Student's *t*‐test for all comparisons. Exact *P* values are shown on the graph.

**Figure 4:**
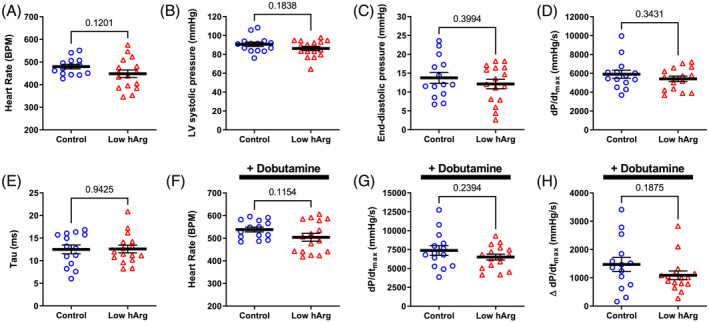
Homoarginine deficiency study: left ventricular (LV) haemodynamics 6.5 weeks after myocardial infarction. (A) Heart rate; (B) LV end‐systolic pressure; (C) LV end‐diastolic pressure; (D) dP/dt_max_ as a measure of contractility; (E) tau—the time constant of isovolumetric relaxation. The remaining panels were measured under maximal β‐adrenergic stimulation via dobutamine infusion: (F) maximal heart rate; (G) maximal dP/dt_max_; (H) contractile reserve measured as change in dP/dt_max_ from baseline. Data are mean ± SEM from *n* = 14 control and *n* = 16 low hArg mice, using unpaired Student's *t*‐test for all comparisons except baseline heart rate where a Welch's correction was used to account for unequal variances. Exact *P* values are shown on the graph.

Mice were given a cine‐MRI 6 weeks after induction of MI (*Figure* *3*). Both groups exhibited hallmarks of chronic heart failure, for example, a large dilated LV, mean ejection fraction <20%, and low cardiac output. However, levels of circulating hArg had no influence on any of the measured imaging outcomes. A few days later, mice were cannulated for direct LV pressure measurements (*Figure* *4*). Again, we observed values consistent with the development of chronic heart failure in both groups, for example, low LV systolic pressure, elevated end‐diastolic pressure, low dP/dt_max_ (a measure of contractility), and prolonged relaxation (Tau). However, low hArg was not associated with worsening of any parameter, even when measured under stress conditions of maximal β‐adrenergic stimulation using dobutamine (*Figure* *4* *F–H*). The ratio of lung weight to tibial length measured post‐mortem was 10.8 ± 4.5 and 9.5 ± 1.6 for WT and KO mice, respectively (*P* = 0.85). Post‐hoc power calculations indicate that this study had 80% power to detect a difference between means of 6.5 ejection fraction units or 2100 mmHg/s for dP/dt_max_. Our findings suggest that low homoarginine levels *per se* do not worsen LV remodelling and dysfunction in this model of post‐MI chronic heart failure.

### Study 2: Homoarginine and creatine‐deficiency in heart failure

**Figure 5:**
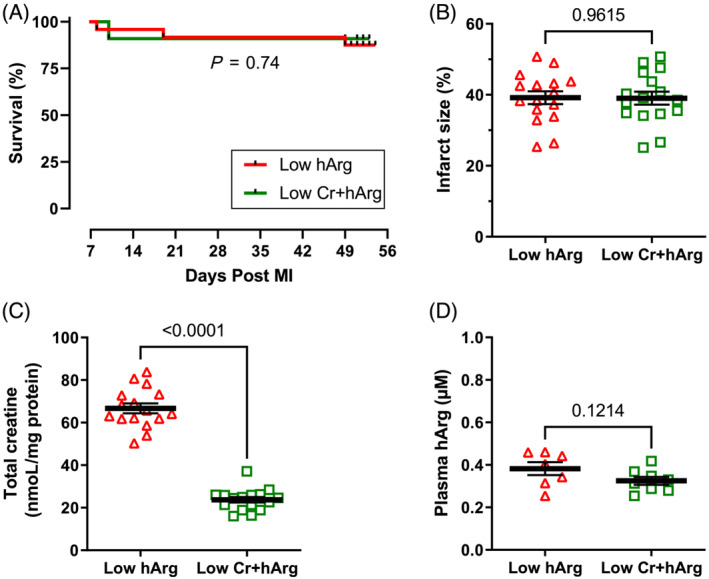
Homoarginine and creatine‐deficiency study: (A) Kaplan–Meier survival curves from 7 days after myocardial infarction in creatine‐fed AGAT knockout mice (low hArg; *n* = 24) and knockout mice where dietary creatine was withdrawn at Day 7 post‐MI (low Cr + hArg; *n* = 22). (B) Surviving mice were matched for infarct size calculated as percentage of LV circumference measured by 3‐D echocardiography. This resulted in *n* = 16 low hArg and *n* = 16 low Cr + hArg for subsequent analysis with a mean infarct size of 39% in both groups. (C) Myocardial total creatine levels were confirmed to be significantly lower in the low Cr + hArg group, whereas both groups had comparable low levels of plasma hArg (D; *n* = 7–8). Data are mean ± SEM with log‐rank test to compare survival curves and unpaired Student's *t*‐test for all other comparisons except for total creatine where a Welch's correction was used to account for unequal variances.

AGAT KO mice were given dietary creatine throughout life, such that all mice had homoarginine deficiency; however, one cohort was changed to creatine‐free diet at Day 7 post‐MI to accelerate loss of myocardial creatine. Survival curves were not significantly different (*Figure* *5* *A*) and are shown from Day 7 because this is the point at which animals were randomized to experimental diets. Survivors from each group were matched for infarct size, which required *n* = 1 low hArg and *n* = 3 low Cr + hArg be excluded, resulting in *n* = 16 mice per group with identical mean infarct size of 39% and a range of 25%–51% (*Figure* *5* *B*). Myocardial total creatine levels were significantly lower in the group switched to a creatine‐free diet, while plasma hArg levels were not significantly different and low in both groups cf. hArg levels in Study 1 (*Figure* *5* *C,D*).

**Figure 6:**
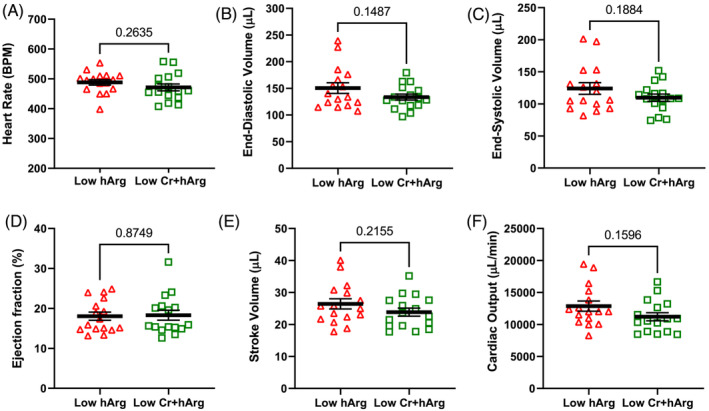
Homoarginine and creatine‐deficiency study: 3‐D echocardiography at 6 weeks after myocardial infarction. (A) Heart rate; (B) left ventricular (LV) end‐diastolic volume; (C) LV end‐systolic volume; (D) ejection fraction; (E) stroke volume; (F) cardiac output. Data are mean ± SEM from *n* = 16 low hArg and *n* = 16 low Cr + hArg mice using unpaired Student's *t*‐test for all comparisons except for Panels (D) and (F) where a Mann–Whitney test was used due to a non‐Gaussian distribution. Exact *P* values are shown on the graph.

**Figure 7:**
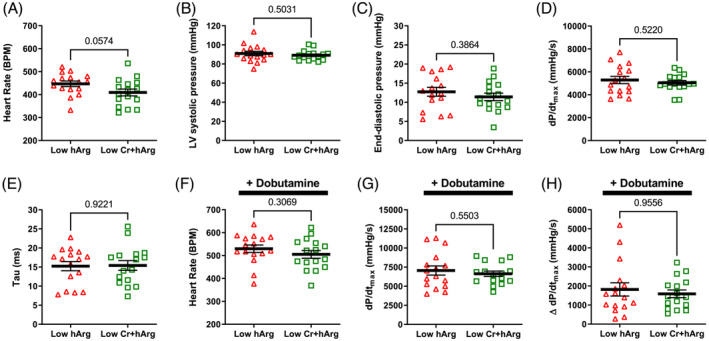
Homoarginine and creatine‐deficiency study: left ventricular (LV) haemodynamics 7.5 weeks after myocardial infarction. (A) Heart rate; (B) LV end‐systolic pressure; (C) LV end‐diastolic pressure; (D) dP/dt_max_ as a measure of contractility; (E) tau—the time constant of isovolumetric relaxation. The remaining panels were measured under maximal β‐adrenergic stimulation via dobutamine infusion: (F) maximal heart rate; (G) maximal dP/dt_max_; (H) contractile reserve measured as change in dP/dt_max_ from baseline. Data are mean ± SEM from *n* = 16 low hArg and *n* = 16 low Cr + hArg mice, using unpaired Student's *t*‐test for all comparisons except for Panels (B) and (G) where a Welch's correction was used to account for unequal variances and Panel (H) where a Mann–Whitney test was used due to a non‐Gaussian distribution. *P* values are shown on the graph.

Mice received a 3‐D echocardiogram six weeks after MI, with both experimental groups exhibiting an overt heart failure phenotype with LV dilatation and grossly impaired ejection fraction. However, no significant differences were observed for any parameter between mice with low hArg levels and those with combined hArg and creatine deficiency (*Figure* *6*). Haemodynamic assessment under baseline and during maximal β‐adrenergic stimulation established values consistent with development of chronic heart failure in both groups, but no significant differences were observed for any parameter between the group with low hArg and the group that also had low creatine levels (*Figure* *7*). The ratio of lung weight to tibial length measured post‐mortem was 8.7 ± 1.2 and 8.2 ± 0.7, respectively (*P* = 0.11). Post‐hoc power calculations indicate that this study had 80% power to detect a difference between means of 4.5 ejection fraction units or 2000 mmHg/s for dP/dt_max_. These findings suggest that the combination of low hArg and low creatine is not detrimental to the chronically failing heart.

## Discussion

Here we show that low circulating levels of hArg do not make a measurable contribution to LV remodelling and dysfunction in a model of surgically induced ischaemic heart failure. Furthermore, that this is true even when combined with low myocardial creatine levels. This suggests that once injury occurs, hArg does not adversely influence disease severity. Note that these findings specifically do not rule out a potential effect of low hArg to influence the likelihood of having an MI in the first instance, or on infarct size, which was controlled for in these experiments in order to purely study the effect on heart failure progression.

The use of AGAT knockout mice is not intended to mimic the rare genetic condition in humans, but as a convenient model that allowed close control of hArg and creatine levels via dietary supplementation. Thus, our findings should be generalizable to patients post‐MI where reduced creatine levels are a universal finding and to the sizeable proportion of patients with naturally low circulating hArg that population studies have shown to be at higher risk of cardiovascular and all‐cause mortality.^1^, ^2^

Our experimental groups achieved levels of hArg and creatine exactly as intended, allowing for a clean and easy to interpret experiment that avoided the confounding effects of life‐long creatine‐deficiency. The only remaining difference was the absence of AGAT protein in knockout animals, which is predominantly expressed in the kidney.^19^ Only one study has described AGAT expression in the heart, which was observed in end‐stage human heart failure, raising the possibility of local creatine (or homoarginine) biosynthesis to support function.^20^ However, we observed no measurable benefit for WT over KO mice in Study 1, arguing against a meaningful role for local creatine and/or hArg biosynthesis in the failing heart.

We have previously described impairment of contractility and relaxation in AGAT KO mice attributable to hArg deficiency, both *in vivo* and in isolated cardiomyocytes.^11^ This is in agreement with the reduction in LV function described in individuals with low circulating hArg within a general population^21^ and led to the hypothesis, tested herein, that pre‐existing dysfunction might be even more detrimental to the failing heart. This was manifestly not the case, which suggests that the influence of low hArg on mortality in population studies is likely linked to modification of cardiovascular disease risk factors rather than a direct effect on heart failure progression. Nevertheless, dietary supplementation of hArg to supra‐physiological levels has been shown to improve haemodynamic indices of LV contractility and relaxation in wild‐type mice with post‐MI heart failure.^22^ Other studies also support the potential for therapeutic hArg supplementation in non‐ischaemic heart failure, for example, due to pressure‐overload^23^ and to protect cardiac function in a rat model of chronic kidney disease.^24^ Hence, low hArg may modify disease risk, while high levels of hArg positively modify disease progression.

Low myocardial creatine levels are a hallmark of the chronically failing heart regardless of aetiology and have long been thought to contribute to cardiac dysfunction.^3^ This thinking persists due to the wealth of correlative data in human heart failure, for example, positive correlations between PCr/ATP ratio and ejection fraction and with survival in patients with dilated cardiomyopathy,^25^, ^26^ but also low PCr/ATP in heart failure with preserved ejection fraction.^27^ Indeed, changes in the CK system that occur in human heart failure are also observed in the mouse and other species where equivalent correlative data exist.^3^, ^6^, ^28^ However, rather than recapitulate a heart failure phenotype, the findings from genetic models of CK system deficiency do not support a causative role. For example, CK knockout models have subtle baseline cardiac dysfunction, but post‐infarct remodelling and heart failure was not any worse.^29^ Creatine‐deficient GAMT KO mice have mild impairment of baseline haemodynamics; however, this did not prove to be detrimental in the setting of chronic heart failure.^8^ A major criticism of these studies is that life‐long deficiencies can result in adaptive compensatory mechanisms, and while adaptive changes were not identified in the GAMT KO,^8^, ^30^ it is impossible to rule them out entirely. Furthermore, we know that GAMT KO mice have potential confounders due to low body weight and the accumulation of the creatine pre‐cursor guanidinoacetic acid, which is also a substrate for CK, albeit with much lower affinity.^7^ The key strength of our current study is the absence of these confounding effects, with no accumulation of creatine pre‐cursors and normal creatine levels maintained throughout development (as evidenced by normal body weights). Importantly, we also test this under conditions of increased work. Hence, this study using AGAT KO represents the strongest evidence to date that low creatine levels *per se* are not detrimental to the failing heart. This agrees with gain‐of‐function studies that used over‐expression of the creatine transporter to maintain higher levels of creatine in the failing heart, which failed to observe any beneficial effects on LV structure or function.^31^

It is an important distinction that myocardial creatine levels have a profound effect on tissue injury following acute MI, which is why we only withdrew dietary creatine 7 days after injury and were careful to match for infarct size. Low levels of creatine increase myocardial injury and impair functional recovery,^7^ while moderately elevated creatine levels are cardioprotective.^31^

### Limitations

It is a limitation that sham‐operated groups were not included in these studies. This was a decision taken for animal welfare reasons, because the baseline in vivo phenotype has already been published for AGAT KO mice.^11^

A further limitation is the use of only female mice, a decision taken because approximately 25% of male C57BL/6J mice die of cardiac rupture in the first week post‐MI,^32^ which adds a significant animal welfare burden, may introduce survival bias, and does not represent the phenotype we wish to study. However, it is important to consider whether this choice of single sex may have influenced outcomes. It is well‐established that ischaemic heart failure is more common in males; however, this study was not concerned with risk factors for disease, but whether hArg and creatine levels influenced established disease. In a human general population, median serum hArg levels were found to be 2.56 and 2.72 μmol/L for women and men, respectively.^33^ This 6% difference is unlikely to be physiologically relevant because a meta‐analysis of 20 published studies confirmed the inverse relationship between circulating hArg and all‐cause mortality, but found that sex did not significantly contribute to the pooled hazard ratio.^34^ Male sex is also known to exacerbate cardiac phenotypes when compared with female mice, which means that experimental groups should be matched for sex (e.g., in CK knockout animals^35^). However, we are not aware of any significant sex differences in terms of myocardial creatine levels or CK expression in normal or heart failure mice. Our previous studies on creatine‐deficient mice used both males and females for baseline cardiac phenotyping, and no sex differences were observed.^8^, ^11^ Therefore, while it is a limitation that males were not included in this study, it seems unlikely that this will have affected the experimental conclusions.

It is also important to consider potential species differences. The tissue expression pattern for creatine and hArg biosynthetic enzymes are comparable between human and mouse,^36^ but mice have lower levels of both circulating hArg and myocardial creatine.^3^, ^33^ This may, in part, reflect species differences in percent muscle mass because skeletal muscle represents the largest turnover of creatine, which will determine AGAT activity via end‐product repression.^37^ However, creatine accumulates in the heart against a concentration gradient, so expression of the creatine transporter, rather than creatine abundance, is a more important determinant.^38^ The reason for lower myocardial creatine in mice has not been established, but it means that the fall in creatine levels that typically occur in murine heart failure is less extreme than those observed in humans and large animal models.^3^ In the current study, we circumvent this by pushing creatine levels even lower by withdrawing dietary creatine in the AGAT knockout mice.

## Conclusions

In conclusion, while creatine or hArg deficiency impairs haemodynamic function in the otherwise healthy heart,^11^ it is clearly not additive to the dysfunction observed post‐MI. Neither low homoarginine nor low creatine levels, alone or in combination, were sufficient to exacerbate chronic heart failure following MI in the mouse. This argues against either metabolite having a major causative role in the development of LV dysfunction post‐MI, although it does not preclude an effect on cardiovascular disease risk factors. Future studies should therefore focus on how hArg modifies disease risk and the mechanisms by which hArg has been shown to influence contractile function. Our findings suggest that it is unnecessary to correct low hArg levels in patients with chronic heart failure, nevertheless, aiming for supra‐physiological levels represents a promising strategy,^22^ which merits further study in patient populations.

## Conflict of interest

None declared.

## Funding

This work was funded by British Heart Foundation Programme Grants (RG/13/8/30266, RG/18/12/34040). Additional core support is acknowledged from the Oxford British Heart Foundation Centre for Research Excellence (RE/18/3/34214) and Wellcome Trust Core Award (Grant No. 203141/Z/16/Z).
